# Distinct cellular responses differentiating alcohol- and hepatitis C virus-induced liver cirrhosis

**DOI:** 10.1186/1743-422X-3-98

**Published:** 2006-11-22

**Authors:** Sharon L Lederer, Kathie-Anne Walters, Sean Proll, Bryan Paeper, Shahar Robinzon, Loreto Boix, Nelson Fausto, Jordi Bruix, Michael G Katze

**Affiliations:** 1 Department of Microbiology, University of Washington, Seattle, WA, USA; 2 Ben-Gurion University, Beer-Sheva, Israel; 3 Barcelona Clinic Liver Cancer Group, Liver Unit, Hospital Clinic IDIBAPS, Barcelona, Spain; 4 Department of Pathology, University of Washington, Seattle, WA, USA

## Abstract

**Background:**

Little is known at the molecular level concerning the differences and/or similarities between alcohol and hepatitis C virus induced liver disease. Global transcriptional profiling using oligonucleotide microarrays was therefore performed on liver biopsies from patients with cirrhosis caused by either chronic alcohol consumption or chronic hepatitis C virus (HCV).

**Results:**

Global gene expression patterns varied significantly depending upon etiology of liver disease, with a greater number of differentially regulated genes seen in HCV-infected patients. Many of the gene expression changes specifically observed in HCV-infected cirrhotic livers were expectedly associated with activation of the innate antiviral immune response. We also compared severity (CTP class) of cirrhosis for each etiology and identified gene expression patterns that differentiated ethanol-induced cirrhosis by class. CTP class A ethanol-cirrhotic livers showed unique expression patterns for genes implicated in the inflammatory response, including those related to macrophage activation and migration, as well as lipid metabolism and oxidative stress genes.

**Conclusion:**

Stages of liver cirrhosis could be differentiated based on gene expression patterns in ethanol-induced, but not HCV-induced, disease. In addition to genes specifically regulating the innate antiviral immune response, mechanisms responsible for differentiating chronic liver damage due to HCV or ethanol may be closely related to regulation of lipid metabolism and to effects of macrophage activation on deposition of extracellular matrix components.

## Background

More than 170 million people worldwide are chronically infected with hepatitis C virus (HCV) [1] and about 20% of those infected will develop cirrhosis [2]. The incidence of cirrhosis development due to chronic alcohol exposure is similar, and half the causes of death following end-stage liver disease are due to this etiology [3,4]. In alcoholic liver disease (ALD), the metabolism of ethanol plays a role in pathogenesis [5-8], and there is increasing evidence for specific roles played by the immune response  [4][5,9-15]. Ethanol can suppress innate immunity in mice by attenuating the TLR3-ds RNA signaling pathway and subsequent interferon (IFN) response [11], as well as in actively drinking patients by decreasing expression of CD28/B7 co-stimulatory pathways [10]. In addition, β-chemokine production and migration of mononuclear cells has been observed during later stages of ALD [13].

Early cirrhosis due to HCV infection can be difficult to recognize, as there are often only nonspecific symptoms associated with its progression. The standard method of determining the severity of disease and appropriate treatment has been the liver biopsy [16], but histological examination of the biopsy gives limited information on what is occurring at the molecular level. Therefore, identifying gene expression patterns in liver tissue during cirrhosis development could add valuable information to clinical tests. Functional genomics is increasingly being utilized to investigate the mechanisms underlying the development of liver disease. During chronic HCV infection in humans, broad activation of the endogenous type I IFN pathway has been observed by genomic analysis [17]. Microarray analysis of liver biopsies from HCV-infected patients has also revealed genes associated with activated lymphocytes, extracellular matrix, and macrophages, that could be possible markers for fibrosis progression[18,19]. In mice, expression of the HCV core protein results in down regulation in the expression of lipid metabolism-associated genes [20]. Other virus-host interactions, including SARS-CoV [21], influenza virus [22], and Ebola [23], have also been successfully studied by functional genomics. Gene expression profiling using microarray technology clearly makes it possible to obtain global transcriptional changes associated with a diseased state caused by many factors, which could help identify pathways implicated in progression of disease [24].

In the current report we utilized functional genomic analysis to compare gene expression patterns in cirrhotic livers, including various Child-Turcotte-Pugh (CTP)-classifications, associated with either chronic alcohol consumption or HCV infection. The pathology of alcohol- and HCV-induced cirrhosis is very similar and characteristic fibrosis patterns leading to cirrhosis can overlap among these two etiologies [25]. Therefore, it is particularly useful to now have the tools for genomic analysis, in order to determine how cirrhosis development may differ between these two distinct etiologies. Upon analysis, we indeed found differences in the expression of interferon-related genes and lymphocyte-specific genes between alcoholic and viral etiologies. Interestingly, a clear distinction existed between CTP classes of ALD, but not HCV-associated, cirrhosis. Different expression patterns depending on severity of alcohol-induced cirrhosis were found in genes associated with the inflammatory response, lipid metabolism, and oxidative stress. This information could be integral in increasing understanding of underlying mechanisms at work during end-stage liver disease.

## Results

### Global gene expression patterns distinguish ethanol- and HCV-induced cirrhosis

**Figure 1:**
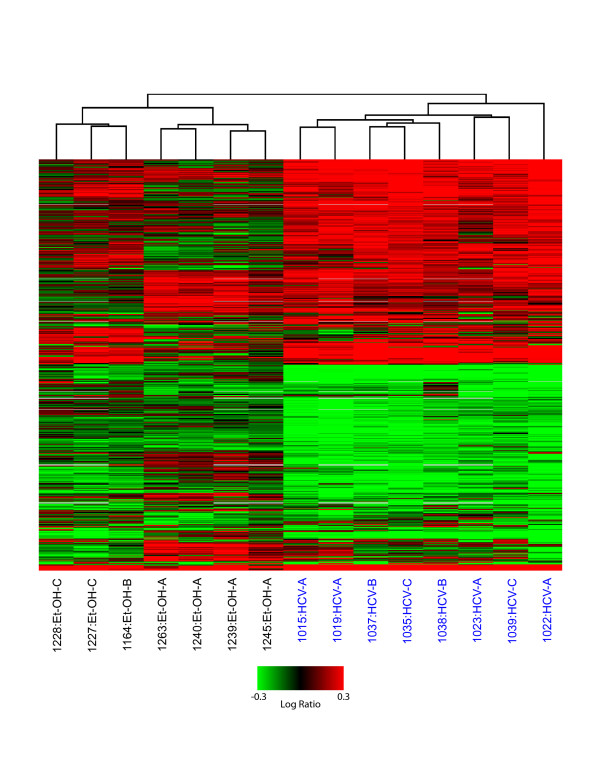
**Global gene expression analysis of liver tissue from HCV and ethanol-associated cirrhosis**. 2-D hierarchical clustering was performed using Resolver System software with agglomerative algorithm, average link heuristic criteria, and Euclidian correlation metric. Each column represents gene expression data from an individual experiment comparing cirrhotic and normal liver and the cluster represents 2965 genes that showed a >2-fold change (P value < 0.05) in at least 3 of 15 experiments. A full view is shown with green and red colors showing decreased or increased levels, respectively, of mRNA expression relative to normal (non-cirrhotic, uninfected) tissue. HCV-infected samples are highlighted in blue.

To analyze gene expression changes in cirrhotic livers associated with HCV (n = 8) or ethanol (n = 7), microarray experiments were performed comparing each type of cirrhotic liver to a reference pool of normal liver tissue. Cirrhosis severity was evaluated according to the CTP clinical scoring system (A-C, least to most severe, respectively) and patient scores are summarized in Table 1. The global gene expression profiles of all experiments are shown in Figure 1. In total, 2,965 genes were differentially expressed (≥ 2-fold, P ≤ 0.05) in at least 3 of 15 samples. The clustering algorithm demonstrates that global gene expression profiles clearly distinguish ethanol- and HCV-associated cirrhotic samples (indicated with black and blue text, respectively). Substantially more gene expression changes were observed in HCV-related samples compared to ethanol-related, ranging from 574–1622 in HCV samples as compared to 79–683 in ethanol samples. Interestingly, the gene expression patterns observed were relatively uniform among HCV-cirrhotic samples and did not appear to differ between CTP classes. In contrast, the global gene expression profiles demonstrated distinct patterns among CTP classes in ethanol-related cirrhosis.

**Table 1:** Patient profile

**Patient #**	**Sex**	**Age**	**Etiology**	**Child-Pugh**	**Length of alcohol consumption**	**Quantity of alcohol consumed**	**ALT/AST**
1239	M	68	Alcohol	A	several years	> 100 g/day	28/37
1240	M	70	Alcohol	A	several years	> 100 g/day	36/44
1245	M	67	Alcohol	A	unknown	unknown	19/19
1263	M	72	Alcohol	A	> 20 years	80 g/day	82/89
1164	M	62	Alcohol	B	> 30 years	> 80 g/day	33/27
1227	M	59	Alcohol	C	39 years active at OLT	> 100 g/day	28/54
1228	M	44	Alcohol	C	18 years	> 100 g/day	18/27
1015	M	47	HCV	A	N/A	none	96/45
1019	M	66	HCV	A	N/A	none	77/99
1022	M	61	HCV	A	N/A	none	113/143
1023	M	67	HCV	A	N/A	none	90/115
1037	F	54	HCV	B	N/A	none	57/78
1038	M	55	HCV	B	N/A	none	69/108
1035	M	59	HCV	C	N/A	none	87/100
1039	M	66	HCV	C	N/A	none	90/35

### Gene expression changes specific to HCV-infected cirrhotic livers are associated with induction of the innate antiviral immune response

**Figure 2:**
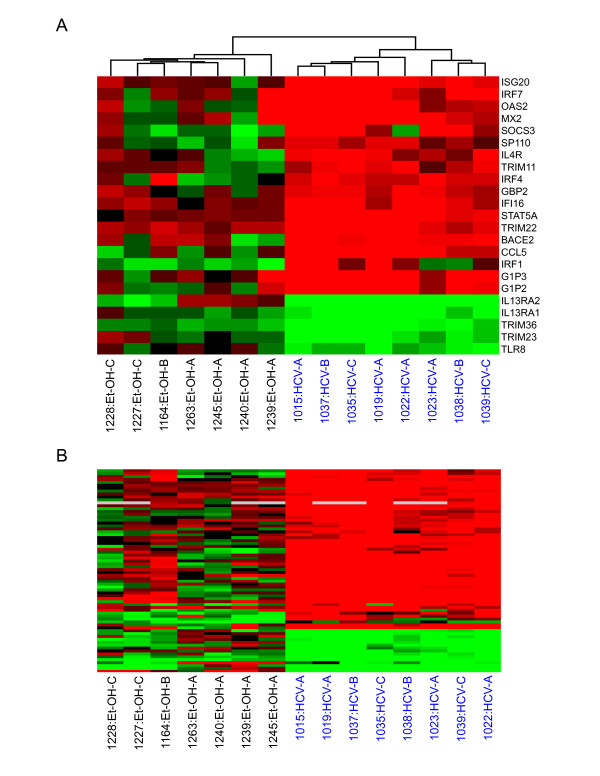
**Differences in global gene expression profiles between HCV and ethanol-associated cirrhotic liver tissue**. **A**. Expression of genes associated with IFN response. HCV- and EtOH-associated cirrhosis samples were clustered based on the expression of IFN-related genes that were at least 2-fold regulated with a *P *value ≤ 0.05 in at least 3 of 15 experiments, comparing cirrhotic tissue to a normal liver reference. See legend of Fig. 1 for details on clustering, the applied color scheme for the fold changes in the mRNA levels, and experiment representation. **B**. Expression of genes associated with lymphocyte activation and proliferation. Cirrhotic samples were again clustered based on the expression of lymphocyte-related genes that were at least 2-fold regulated with a *P *value ≤ 0.05 in at least 3 of 15 experiments comparing cirrhotic tissue to a normal liver reference. See legend of Fig. 1 for details on clustering, the applied color scheme for the fold changes in the mRNA levels, and experiment representation. See Supplementary Table 1 for a list of gene names represented in Figure 2B.

To further classify functional pathways specifically associated with etiology, we performed a one-way error-weighted ANOVA comparison of HCV- and ethanol-associated cirrhotic liver biopsies. A set of 946 genes was identified as being significantly (P ≤ 0.05) different with respect to expression level between the two groups. We then used Fatigo software to analyze functional groups that differed between cirrhosis etiologies. Genes associated with metabolism, the immune response, and cell death were differentially regulated in HCV- and alcohol-related cirrhosis. Many of the genes specifically induced in HCV-associated cirrhotic livers were not up-regulated in ethanol-cirrhotic livers. This appears to be at least partly related to activation of the innate antiviral immune response, as many of these up-regulated genes are known to be regulated by IFN (Fig. 2A). Specifically, a subset of genes highly induced in HCV-associated cirrhotic samples were IFNα/β-inducible (IRF7, OAS2, GIP2, GIP3) or take part in its activation. Additional IFN-type I genes – OAS1, IFITM1, IFI35, IFIT1, MX1, IFI27 – were highly up-regulated in HCV-associated samples but not included in this ANOVA. A small set of genes (ISG20, IFI16, TRIM22, STAT5A) were also highly up-regulated in nearly all samples, including ethanol-cirrhotic livers, suggesting these may play a role in an inflammatory response related to cirrhosis development regardless of etiology.

Significant infiltration of lymphocytes into the liver is observed during chronic HCV infection [26]. In this study, up-regulation of gene markers for lymphocytes was indeed much more pronounced in HCV-infected liver samples than in ethanol-induced livers (Fig. 2B, Additional file 1). Specifically, RANTES, which is chemotactic for T cells *in vitro *was significantly up-regulated in all HCV-infected livers (1.8–4.5 fold, P ≥ 0.05). In contrast, circulating levels of natural killer cells have been shown to be suppressed under the influence of alcohol [27,28]. Consistent with this, we observed decreased expression of natural killer cell-related genes, including CD44, IL15R, CD2 and CCL5, in alcohol-associated cirrhotic liver samples.

### Gene expression patterns differentiate ethanol-associated cirrhosis by CTP class

**Figure 3:**
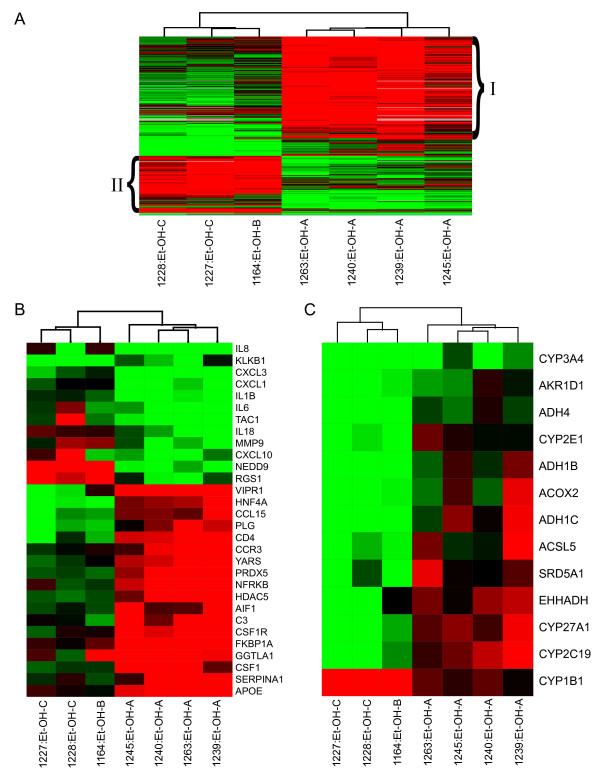
**Differences in global gene expression profiles between different CTP classes of ethanol-associated cirrhotic liver tissue**. **A**. Expression profiles of genes differentially expressed in ethanol-related cirrhosis CTP class A versus CTP BC (identified from one-way ANOVA). Each column represents gene expression data from an individual experiment comparing cirrhotic and normal liver and the cluster represents genes that showed a >2-fold change (P value < 0.05) in at least 3 of 7 experiments. See legend of Fig. 1 for details on clustering, the applied color scheme for the fold changes in the mRNA levels, and experiment representation. Grouping (I and II) is discussed in the text and demonstrates differences between CTP classes in gene expression associated with various biological processes. Refer to Supplementary Table 2 for gene names in each group. **B**. Expression of genes associated with the inflammatory response. Cluster depicts expression of genes that were at least 2-fold regulated with a *P *value ≤ 0.05 in at least 3 of 7 experiments comparing cirrhotic tissue to a normal liver reference. See legend of Fig. 1 for details on clustering, the applied color scheme for the fold changes in the mRNA levels, and experiment representation **C**. Expression of genes involved in fatty acid and bile acid metabolism. Expression of genes that were at least 2-fold regulated with a *P *value ≤ 0.05 in at least 3 of 15 experiments comparing cirrhotic tissue to a normal liver reference. See legend of Fig. 1 for details on clustering, the applied color scheme for the fold changes in the mRNA levels, and experiment representation.

Whereas HCV-associated cirrhotic samples looked very similar regardless of CTP class (Fig. 1), global gene expression profiles indicated that CTP class A ethanol-associated samples exhibited unique expression patterns from CTP class B and C samples. In order to further identify which gene expression patterns were specific depending on severity of ethanol-associated cirrhosis, we performed a one-way error-weighted ANOVA (P ≤ 0.05) of less severe (CTP class A) cirrhotic samples versus more severe (CTP classes B and C) samples (Fig. 3A, Additional file 2). Expression profiles distinguished the ethanol-related CTP class A samples from ethanol-related CTP class B and C samples. In general, class A samples demonstrated up-regulation of more genes than in samples with more severe cirrhosis (Class B and C). Distinct gene expression profiles were not observed between CTP classes B and C, but this may be due to small sample size. Ingenuity analysis of groups I and II (Fig. 3A) demonstrated that the number of genes associated with various biological functions differed significantly depending on severity of alcohol cirrhosis. Of 135 annotated genes up-regulated in group II, 60 were associated with cell death as compared to only 16 in group I. High numbers of genes related to cell movement, mainly of phagocytes, and tissue development were also up-regulated in more severe ethanol cirrhosis. Many genes in group I (less severe cirrhosis) were associated with lipid metabolism, specifically the metabolism of cholesterol. Genes involved in the function of the immune and lymphatic system, such as activation of mononuclear leukocytes and proliferation of macrophages, were also up-regulated in early, less severe cirrhosis.

A number of genes that were nearly exclusively up-regulated in the CTP-A class of ethanol cirrhosis included inflammatory-related genes (Fig. 3B). Some of these – VIPR1, CCL15, PLG, CCR3, CSF1 – are involved in macrophage activation and migration. Macrophage-related genes not in this ANOVA set but also highly up-regulated in CTP class A samples included FN1, C5, LPA, and SERPINA3.

Interestingly, severe ethanol cirrhosis differed from less severe ethanol cirrhosis in expression of genes associated with fatty and bile acid metabolism (Fig. 3C). Many of these genes were up-regulated during less severe cirrhosis but down-regulated by end-stage ethanol cirrhosis. Sterol 27-hydroxylase (CYP27A1) oxidizes cholesterol in the alternative pathway of bile acid biosynthesis; a defect in this enzyme leads to excessive accumulation of sterols [29]. We observed significant up-regulation of this gene in livers of all patients with early ethanol cirrhosis and, as with other genes in this group, down-regulation during late (end-stage) cirrhosis (Figure 3C). Fatty acids are substrates for cytochrome CYP2E1 (P450 2E1) [5], which is a key enzyme in the microsomal ethanol oxidation system and plays a role in alcohol detoxification and nutritional support [30]. It is also a major source of oxidative stress [6,5], and both protein and mRNA levels increase dramatically in actively drinking patients [31]. Therefore it was interesting that significant down-regulation of this gene was also observed in high grade (CTP class B and C) cirrhotic livers (Figure 3C). A similar pattern was observed for genes encoding alcohol dehydrogenases (ADH4, ADH1B, ADH1C).

### Differential gene expression in ethanol-induced class A cirrhosis

**Figure 4:**
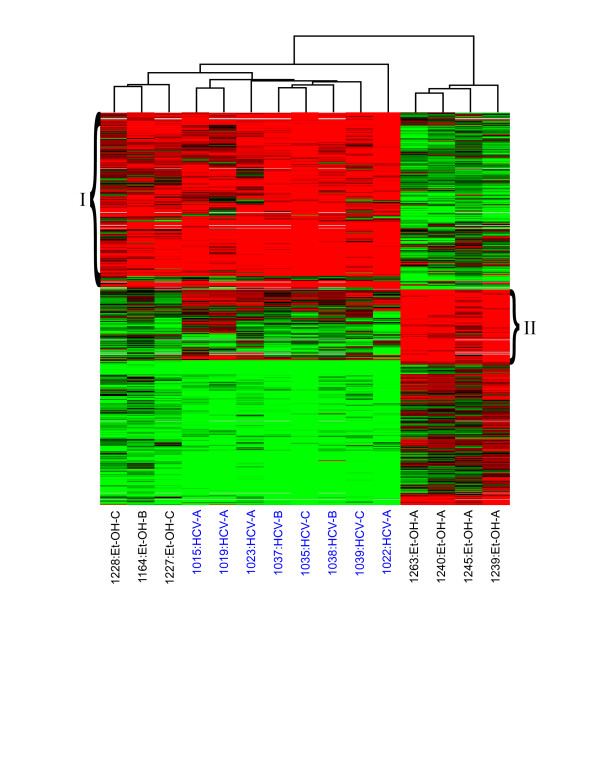
**Differences in global gene expression profiles between ethanol-induced (Class A) cirrhotic tissue versus HCV (Class A, B, C) and ethanol (Class B and C)-induced cirrhotic tissue**. Expression profiles of genes differentially expressed in early ethanol-related cirrhosis versus all other samples; as identified by ANOVA. Each column represents gene expression data from an individual experiment comparing cirrhotic and normal liver and the cluster represents genes that showed a >2-fold change (P value < 0.05) in at least 3 of 15 experiments. See legend of Fig. 1 for details on clustering and the applied color scheme. Refer to Supplementary Table 3 for names of genes represented in this figure.

CTP class A alcohol-associated samples appeared to have unique expression profiles compared to all other samples regardless of etiology (Fig. 1). We therefore performed a one-way error-weighted ANOVA (P ≥ 0.05) of less severe (CTP class A alcohol-associated) versus more severe ethanol-related and HCV-associated cirrhotic samples to identify genes with expression levels unique to CTP class A alcohol. Of 607 total genes in this set, a cluster of 273 were highly up-regulated in all samples but ethanol CTP class A (Fig. 4, group I), and a cluster of 109 were highly up-regulated specifically in class A (Fig. 4, group II); see Additional file 3 for more information on gene names. Functional analysis (Ingenuity) revealed that genes associated with cell death, movement, and the immune response were much more abundant in group I than in group II. Most immune response genes up-regulated in group I were generally involved in proliferation and migration of leukocytes, while those in group II were again related to activation and infiltration of macrophages. The majority of genes up-regulated in group II of early ethanol cirrhosis are involved in the modification of lipids. The response to oxidative stress was also significantly different in early ethanol cirrhosis as related to all other samples. Two genes in particular were highly up-regulated in CTP class A livers – PRDX2 (up to 4.7 fold) and APOE (up to 17.5 fold) – and either less up-regulated or even down-regulated in the majority of the remaining samples.

## Discussion

Global gene expression profiling of liver biopsy material revealed significant differences between HCV and ethanol-induced cirrhosis. In general, gene expression changes in cirrhotic livers are more numerous in HCV-infected livers compared to alcohol-induced diseased livers. Gene expression patterns can also distinguish classes of ethanol-associated cirrhosis. Additionally, unique differences in gene expression existed between early ethanol cirrhosis and all other liver samples.

The majority of these differences between HCV and ethanol etiologies involved the expression of genes associated with the immune response, including the IFN response and infiltration of immune cells. This suggests that the immune response may play a more prominent role in viral versus ethanol-induced cirrhosis. Our data showing significant up-regulation of IFN-type I genes specifically in HCV-associated samples are also consistent with previous studies that demonstrated activation of innate antiviral signaling pathways during HCV infections in chimps [32] and humans [17,18,26,33-37].

In this report, gene markers for lymphocytes were also very pronounced in HCV-infected livers. Increased expression of RANTES – chemotactic for T cells – has been observed in livers of chronically-infected HCV patients [38] and a correlation has been found between increased expression of RANTES in the liver and the extent of liver damage [39]. That we observed significant up-regulation of genes related to lymphocyte infiltration exclusively in HCV-infected cirrhotic livers may have some significance for how liver injury differs in HCV- and ethanol-cirrhosis. It has already been shown that liver injury during chronic HCV infection is associated with increased expression of TH1 cytokines [40,41]. However, T-cell activation is also seen in patients during chronic alcohol consumption [42]. The present study points to a predominant role of the immune response in liver pathogenesis well into the latest stages of HCV-associated disease, and T cells seem to be important in this response. Though the activation of T cells appears to contribute to liver injury in chronic ALD, our observations suggest that the immune response could be more important in the stages before cirrhosis develops, but no longer predominant during cirrhosis progression.

Significant differences in gene expression patterns between classes of ethanol-associated liver cirrhosis point to the role of macrophages in progression of ethanol-induced liver disease. Macrophages and other cells of the innate immune system have already been implicated in liver injury during alcoholic liver disease [43-47,12-14]. Induction of cytokine production by Kupffer cells activates hepatic stellate cells, leading to excess accumulation of extracellular matrix and fibrosis development. Production of cytokines and reactive oxygen species from monocytes has been shown to increase in response to chronic alcohol intake but decrease with acute alcohol intake [43]. We found an increase in the expression of macrophage-related genes in early less severe (CTP A) ethanol cirrhosis, but not in late more severe (CTP B and C) ethanol cirrhosis. Though sample number does not allow for strictly defining less or more severe cirrhotics, the trend observed may have some significance related to regulation of metabolism.

Genes specifically linked to metabolism also demonstrated differential regulation depending on cirrhosis stage in alcoholic liver disease. The metabolism of alcohol leads to fat accumulation and oxidative stress which, through the activation of hepatic stellate cells, eventually leads to liver fibrosis [48]. As the CYP system is the most important enzyme system for drug metabolism, it is interesting that several genes encoding these proteins were up-regulated only in livers exhibiting early ethanol cirrhosis. CYP27A1, an oxidizer of cholesterol, and CYP2E1, a key enzyme in the microsomal ethanol oxidation system, were both up-regulated in most early (CTP class A) cirrhosis samples but significantly down-regulated by more severe, end-stage cirrhosis. As CYP27A1 is an acute phase gene repressed by lipopolysaccharide and cytokines, it is possible that the down-regulation of this gene is linked to levels of cytokine activity during late-stage ethanol cirrhosis [49].

We know that Kupffer cells harbor CYP2E1 [50], become active in ALD, and produce inflammatory cytokines [4]. It could therefore be consistent that we observe both an increase in expression of macrophage-related genes as well as genes involved in cholesterol oxidation at the same (early) stages of cirrhosis. Macrophages may be an important source for lipid metabolism enzyme activity at the beginning stages of cirrhosis, but become less necessary as there is decreased demand for active metabolism of alcohol. Again, this is only speculation as we realize that sample size is limiting our ability to definitively group CTP class B with CTP class C as late/severe cirrhotics. Alternatively, this down-regulation of genes during later stages of ethanol cirrhosis may reflect widespread cell death and shut-down of liver function.

It is interesting that there were no significant changes in gene expression patterns observed between classes of HCV-induced cirrhotic livers. This may be evidence for a uniform host response throughout cirrhosis development in this etiology. However, it is more likely that the similarities seen among HCV-infected livers are due to the antiviral host response to HCV infection.

## Conclusion

It is clear that gene expression profiles in ethanol-induced and HCV-induced liver cirrhosis are distinct. We have identified functional groups that differ both between etiologies and within ethanol-induced cirrhosis, depending on the extent of liver damage. The data suggest that oxidative stress, lipid metabolism, and macrophage activity may be more associated with severe liver injury leading to cirrhosis in ALD than in viral-induced disease. In contrast, the adaptive immune response, characterized by T cell-mediated killing of HCV-infected cells, appears to be more associated with the development of liver cirrhosis in viral-, but not alcohol-mediated disease. The study supports the idea that though these different etiologies lead to similar pathologies, the cellular mechanisms responsible for disease progression may be unique. Although we realize the limitations of a small cross-sectional study, the gene expression patterns identified here are from a large set of genes in order to give a broad global perspective. These initial findings will contribute to further studies to distinguish which pathways can differentiate development of liver damage due to etiology, which could lead to better-targeted therapies for end-stage liver disease.

## Methods

### Human Liver Tissue Samples

Cirrhotic liver samples from 7 alcoholic and 8 HCV-infected patients were obtained from liver disease patients at the University of Barcelona, Spain who underwent liver transplant or hepatic resection. None of the HCV-infected patients in this study were undergoing anti-viral treatment at the time the liver sample was obtained. Alcohol consumption information was based on data obtained in patient interviews. The severity of cirrhosis was clinically graded according to Child-Turcotte-Pugh (CTP) scores (summarized in Table 1). Normal, uninfected liver tissue from 8 patients who underwent tumor resection due to metastases of non-liver origin was collected at the same clinic (non-tumor material was used). These uninfected samples were pooled to create a standard normal liver reference (pNL) that was used for all microarray experiments. All patients gave informed prior consent to protocols approved by the University of Barcelona, Spain.

### Total RNA Isolation and mRNA Amplification

Frozen liver tissues were disrupted in Trizol Reagent (Invitrogen, Carlsbad, CA) using a Polytron homogenizer (PowerGene 700, Fisher Scientific), and total RNA was isolated according to the Trizol protocol. All total RNA samples were double amplified using the RiboAmp RNA Amplification kit (Arcturus, Mountain View, CA). The quality of amplified RNA was evaluated by capillary electrophoresis using an Agilent 2100 Bioanalyzer (Agilent Technologies, Palo Alto, CA).

### Expression Microarray Format and Data Analysis

Microarray format, protocols for probe labeling, hybridization, slide treatment, and scanning were performed as previously described [35] and are also available at our website [51]. Human cDNA set 1 expression arrays carrying 13,026 unique cDNA clones were purchased from Agilent. A single experiment comparing two samples, one from a liver with infection and one from the normal liver reference, was performed with four replicate arrays using the dye label reverse technique as described before, thus providing mean ratios between the expression levels of each gene in the analyzed sample pair, standard deviations, and *P *values [35,52-54]. All data were entered into a custom-designed database, Expression Array Manager, and then uploaded into Rosetta Resolver System 4.0 (Rosetta Biosoftware, Kirkland, WA) and Spotfire Decision Suite 7.1.1 (Spotfire, Somerville, MA). Data normalization and the Resolver System Error model specifically developed for this array format are described on the web site [51]. This web site is also used to publish all primary data in accordance with the proposed standards [55]. The annotation sites Fatigo [56] and Ingenuity [57] were used to classify genes into functional groups.

## Supplementary Material

Additional File 1 "Genes differentially regulated in Figure Figure 2B" The table lists gene names for heat map shown in Figure 2B. Click here for file

Additional File 2 "Goup I and II gene names for Figure 3A" The table list gene names for biological function groups in Figure 3A. Click here for file

Additional File 3 "Group I and II gene names for Figure 4" The table lists gene names for groups differentiating cirrhosis class in Figure 4. Click here for file
